# Contamination and Antimicrobial Susceptibility Testing of *Staphylococcus aureus* Isolated from Pork in Fresh Markets, Nongchok District, Thailand

**DOI:** 10.1155/2021/6646846

**Published:** 2021-03-08

**Authors:** Doungjit Kanungpean, Shinji Takai, Tsutomu Kakuda

**Affiliations:** ^1^Faculty of Veterinary Medicine, Mahanakorn University of Technology, 140 Cheum-Sampan Rd., Nongchok, Bangkok 10530, Thailand; ^2^Laboratories of Animal Hygiene, School of Veterinary Medicine, Kitasato University, Higashi 23-351, Towada, Aomori 034-8628, Japan

## Abstract

We surveyed *Staphylococcus aureus* contamination in 110 pork samples from 12 fresh meat markets in Nongchok district, Bangkok, Thailand, and performed antimicrobial susceptibility testing with the disk diffusion method. The prevalence of *S*. *aureus* was 28.18%, and 52 strains were isolated. Antimicrobial susceptibility testing using the disk diffusion method revealed that 80.77% of the isolates were resistant to tetracycline and 76.92% to ampicillin. All strains were 100% susceptible to cloxacillin, cefoxitin, gentamicin, and cefazolin. The high percentage of antibiotic resistance to tetracycline and ampicillin was attributed to their use in treating infections in farmed animals and their addition to animal food for disease prevention. Interestingly, the present study revealed the intermediate resistance of *S*. *aureus* (13.46% of *S*. *aureus*-positive pork samples) to vancomycin which is a common medicine for treating severe infection in humans, suggesting that the trend of resistance might increase and becoming a serious problem of public health for both humans and animals.

## 1. Introduction

*Staphylococcus aureus* is a Gram-positive coccus bacterium that tends to be arranged in grape-like clusters. This bacterium can grow in a medium containing up to 10% salt and is coagulase-positive [1]. *S*. *aureus* is found on the skin and mucous membranes of animals and humans and is also an environmental contaminant [2]. However, in developing countries such as Thailand, where meat is sold at fresh meat markets, the meat may be contaminated by organisms such as *S*. *aureus*, *Escherichia coli,* and *Campylobacter jejuni* [2–4]. *S*. *aureus* is the most common bacterial infection in humans and animals [1]. It can colonize humans and is the leading cause of bacteremia, infective endocarditis, and skin and soft tissue disease (e.g., impetigo and folliculitis); *S*. *aureus* also causes invasive infections and toxin-mediated diseases [1, 5]. Multiple antimicrobial-resistant pathogenic bacteria have been recognized by the World Organization for Animal Health, the Food and Agriculture Organization, and the World Health Organization as an important public health issue [6, 7]. Furthermore, *S*. *aureus* has shown antimicrobial resistance in human and animal therapy [3], and strains of methicillin-resistant *S*. *aureus* (MRSA) are important human pathogens. The spread of MRSA has largely focused on pigs because of widespread worldwide reports of MRSA colonization and antimicrobial resistance in pigs [4]. Our study aimed to determine the extent of *S*. *aureus* contamination and the antimicrobial susceptibility of subsequent isolates in pork sold in fresh meat markets in Thailand.

## 2. Materials and Methods

### 2.1. Sample Collection and *S*. *aureus* Identification

Hundred and ten pork samples were collected randomly for analyzing from 12 markets in Nongchok district, Bangkok, Thailand. Sample amounts of the market no. 1 to no. 12 were 5, 20, 20, 10, 5, 5, 5, 5, 5, 10, 5, and 15, respectively. The samples were kept at 4°C before processing for culture. Each sample was cut into small pieces, placed in approximately 90 ml of buffered peptone water (Himedia Laboratories, Mumbai, India) and mixed for 3 min. Then, 10 *μ*l of the suspension was plated onto Baird-Parker agar (Himedia Laboratories, Mumbai, India) and incubated at 37°C for 18–24 hr. *S*. *aureus* grows as a black and shiny colony, with a fine white rim surrounded by a clear zone on Baird-Parker agar. All colonies identified as *S*. *aureus* were confirmed by a positive result for coagulase [2, 8].

### 2.2. Antimicrobial Susceptibility Testing

Disk diffusion antimicrobial susceptibility tests were performed on the confirmed *S*. *aureus* isolates. The common used antibiotics for treating respiratory disease in farm animals were chosen for the antimicrobial susceptibility tests, including ampicillin (10 *μ*g), cloxacillin (5 *μ*g), cefoxitin (30 *μ*g), cefazolin (30 *μ*g), gentamicin (10 *μ*g), vancomycin (30 *μ*g), and tetracycline (30 *μ*g) (Oxoid Ltd., Hampshire, UK). Briefly, *S*. *aureus* colonies were picked and subcultured by streaking on nutrient agar (Himedia Laboratories, Mumbai, India) and incubating the plate at 37°C for 18–24 hr. Then, a sterilized loop was used to pick four or five colonies from the nutrient agar and they were suspended in 2 ml of Mueller-Hinton broth (Difco Laboratories, Inc., NJ, USA). After adjusting to a turbidity of 0.5 McFarland standards, a sterile swab was dipped into the tube and streaked on Mueller-Hinton agar (Difco Laboratories, Inc., NJ, USA). Then, the appropriate antimicrobial-impregnated disks were placed on the surface of the agar, and the plate was incubated at 37°C for 18–24 hr. Thereafter, we measured the zones of inhibition and determined the antimicrobial susceptibility of the isolates using interpretative standards for *Staphylococcus* species. The results were reported as susceptible, intermediate, or resistant [8, 9].

## 3. Results

Table 1 presents the prevalence of *S*. *aureus* contamination in pork sampled from each fresh meat market. *S*. *aureus* was detected in 28.18% (31/110) of the samples from the 12 markets, including 40% (2 of 5, no. 1 market), 25% (5/20, no. 2 market), 35% (7/20, no. 3 market), 50% (5/10, no. 4 market), 0% (0/5, no. 5 market), 20% (1/5, no. 6 market), 0% (0/5, no. 7 market), 0% (0/5, no. 8 market), 40% (2/5, no. 9 market), 40% (4/10, no. 10 market), 20% (1/5, no. 11 market), and 26.67% (4/15, no. 12 Market). The antimicrobial susceptibility tests are presented in Table 2. Of the 52 isolates tested, 92.23% (49/52) were resistant to one or more antibiotics. Resistance to tetracycline and ampicillin was detected in 80.77% (42/52) and 76.92% (40/52) of isolates, respectively. Resistance to two drugs was found in 67.3% (35/52) of isolates. Furthermore, the *S*. *aureus* isolates were 100% (52/52) susceptible to cloxacillin, cefoxitin, gentamicin, and cephazolin. Additionally, 13.46% (7/52) of isolates were scored as intermediate to vancomycin.

## 4. Discussion

In this study, the prevalence of *S*. *aureus* contamination in pork from 12 fresh meat markets was 28.18%. One of the possibility factors involved in the contamination is “the processing of meat in slaughterhouse” which includes many steps of risk to expose microorganisms such as washing the carcass and portioning of carcass using cutting equipment. The second possibility of contamination is “the locations of fresh meat markets” in the present study which are open area and being nearby streets, car parking, and car transportation with heavy traffic. A recent study reported the occurrence of pathogenic bacterial strains in the environment at a high probability such as *S*. *aureus* which can survive under unfavorable conditions on dry surfaces for a long period, even several months [10]; therefore, the contamination of equipment and hygiene (such as a knife, cutting board, apron, and hand towel) in the slaughterhouse and of fresh meat in the open air markets might occur. A previous study of bacterial contamination's prevalence in similar location to this study supports our finding. A survey reported sushi and sashimi from the flea market at Rangsit Campus, Thailand, were contaminated with *S*. *aureus* 46.88%, that is, a high level of contamination [11] indicating that an open area such as fresh markets or flea markets has a chance to contaminate the pork or cooked foods. Furthermore, the consumer should clean meat before cooking and avoid eating raw meat. Another factor of bacterial contamination is the weather condition of collected sample's location. Our study investigated the pork sample in Bangkok, Thailand, which is a country of hot and humid climate, and the prevalence of *S*. *aureus* contamination was 28.18%. In contrast, a previous study in the USA reported the percentage of pork at 43.3% which is much higher than this study. The integrated geography effects to the prevalence of *S*. *aureus* contamination [12].

*S*. *aureus*, a most common cause of food-borne disease, is a problem of the heath care management due to the resistance to antibiotic toward many classes of antimicrobials [13]. *S*. *aureus* is a leading cause of community-acquired and healthcare-associated bacteremia. Furthermore, *S*. *aureus* causes nosocomial infections in hospitalized patients. Tetracycline and ampicillin are effective against many Gram-positive and Gram-negative bacteria and are used to treat chronic respiratory diseases [4, 14]. However, the antimicrobial susceptibility test results of isolated *S*. *aureus* in our study showed a high percentage of tetracycline- and ampicillin-resistant strains similar to the previous study in pork isolates, which were highly resistant to tetracycline and ampicillin 94.8% and 100%, respectively [10]. Reflecting the limited therapeutic value of these antimicrobial agents for treatment of *S*. *aureus* infected humans [2]. Furthermore, 13.46% of the isolates in our study were intermediate resistant to vancomycin, a drug of choice for treatment of MRSA (methicillin-resistant *S*. *aureus*) infections in hospitals [14]. This prevalence suggests that the level of resistance of *S*. *aureus* to vancomycin might increase in the future in Thailand, resulting in a serious global problem when treating MRSA infections in hospitals [15]. Moreover, recently in India, VRSA (vancomycin-resistant *S*. *aureus*) was already detected 16% from buffalo nasal and skin samples using the disk diffusion method which is a dangerous public health issue [16].

## Figures and Tables

**Table 1:** Prevalence of *Staphylococcus aureus* contamination in pork from each fresh meat market.

No. of markets	Percentage (no. of positive total)
1	40 (2/5)
2	25 (5/20)
3	35 (7/20)
4	50 (5/10)
5	0 (0/5)
6	20 (1/5)
7	0 (0/5)
8	0 (0/5)
9	40 (2/5)
10	40 (4/10)
11	20 (1/5)
12	26.67 (4/15)
**Total**	**28.18 (31/110)**

**Table 2:** Antibiotic susceptibility pattern of *S*. *aureus* isolated from pork from 12 fresh meat markets.

Antimicrobial	Resistant (no. of positive/total isolates)	Intermediate (no. of positive/total isolates)	Susceptible (no. of positive/total isolates)
Tetracycline	80.77 (42/52)	1.92 (1/52)	17.31 (9/52)
Cloxacillin	0	0	100 (52/52)
Cefoxitin	0	0	100 (52/52)
Gentamicin	0	0	100 (52/52)
Ampicillin	76.92 (40/52)	0	23.07 (12/52)
Cephazolin	0	0	100 (52/52)
Vancomycin	0	13.46 (7/52)	86.53 (45/52)

## Data Availability

Previously reported (antimicrobial-resistant strain) data were used to support this study and are available at (DOI: 10.4315/0362-028X-72.3.608, DOI: 10.1111/jfs.12589). These prior studies are cited at relevant places within the text as references [2, 4].
